# Cancer Stem Cell Markers in Head and Neck Squamous Cell Carcinoma

**DOI:** 10.1155/2013/319489

**Published:** 2013-03-03

**Authors:** Aidan G. Major, Luke P. Pitty, Camile S. Farah

**Affiliations:** ^1^School of Dentistry, The University of Queensland, 200 Turbot Street, Brisbane, QLD 4000, Australia; ^2^The University of Queensland Centre for Clinical Research, Herston, QLD 4029, Australia

## Abstract

Head and neck squamous cell carcinoma (HNSCC) is one of the world's top ten most common cancers. Current survival rates are poor with only 50% of patients expected to survive five years after diagnosis. The poor survival rate of HNSCC is partly attributable to the tendency for diagnosis at the late stage of the disease. One of the reasons for treatment failure is thought to be related to the presence of a subpopulation of cells within the tumour called cancer stem cells (CSCs). CSCs display stem cell-like characteristics that impart resistance to conventional treatment modalities and promote tumour initiation, progression, and metastasis. Specific markers for this population have been investigated in the hope of developing a deeper understanding of their role in the pathogenesis of HNSCC and elucidating novel therapeutic strategies.

## 1. Introduction

HNSCC is the eighth and 13th most common malignancy in the world for males and females, respectively, with the majority of malignancies of the upper aerodigestive tract being oral squamous cell carcinomas [1–5]. Despite advances in the understanding and treatment of HNSCC, survival rates have not significantly improved for over 30 years, with the five-year survival rate after diagnosis remaining at 15–50% [1, 6–8]. Current treatments for HNSCC can be traumatic, painful, and disfiguring, drastically affecting quality of life [9–11]. At present, management of HNSCC includes surgical resection and/or combination chemotherapy and radiation therapy [2, 4, 6]. Despite these treatments, the prognosis of HNSCC remains poor due to late stage diagnosis, high rates of primary-site recurrence, and common metastases to locoregional lymph nodes [1, 3, 4, 6, 12]. A desire to improve diagnostic capabilities and treatment efficacy has led to a need for a better understanding of the pathogenesis and characteristics of HNSCC. 

Observations of the initiation, progression, and recurrence of cancer have led to two main hypotheses. The stochastic model suggests that there is an accumulation of numerous and varied individual mutations and microenvironmental signals that provide a selective advantage to certain tumour cells however all tumour cells have the ability to propagate the tumour [13, 14]. The probability of the necessary mutations in any given individual cell is very low [13, 15]. Conversely, the cancer stem cell hypothesis proposes a hierarchical model of tumour initiation and progression which suggests that only a specific subpopulation of self-sustaining cancer cells have the exclusive ability to maintain the tumour [7, 13, 16, 17]. Recent research suggests that part of the mechanisms of recurrence and metastases in some cancers may be due to cancer stem cells (CSCs) [7, 15, 16, 18]. There is mounting evidence for the presence of CSCs in HNSCC with dramatic implications for diagnosis, prognosis, and treatment.

## 2. The CSC Concept 

CSCs are defined as a small subpopulation of cancer cells that constitute a pool of self-sustaining cells with the exclusive ability to cause the heterogeneous lineages of cancer cells that comprise the tumour [7, 17, 19]. There are three main characteristics of CSCs. Initially, the cell must show potent tumour initiation in that it can regenerate the tumour which it was derived from a limited number of cells. In addition, the cells should demonstrate self-renewal *in vivo*, which is practically observed via regrowth of phenotypically indistinguishable and heterogeneous tumours following serial transplantation of reisolated CSCs in secondary and tertiary recipients. Finally, the cells must show a differentiation capacity, allowing them to give rise to a heterogeneous progeny, which represents a phenocopy of the original tumour [15, 17].

Although some have argued that CSCs may arise not only from normal stem cells by mutation of genes that render the cells cancerous but also from progenitor cells, it is hypothesised that these cells experience further genetic alterations and therefore become dedifferentiated and acquire CSC features [17, 18]. The term cancer stem cells refers to the functional properties of the cells and not their origin [19, 20].

In 1978, Julius Cohnheim proposed the concept that tumours were the result of residual embryonic cells [21]. Almost two decades later, a CD34^+^CD38^−^ subpopulation of cells were isolated in acute myeloid leukaemia which, when transplanted into NOD/SCID mice, were capable of initiating acute myeloid leukaemia [22]. Then in 2003, the first solid tumour CSCs were isolated from breast cancer using a CD44^+^CD24^−^Lin^−^ marker phenotype [23]. To date, CSCs have been isolated in hematopoietic malignancies and several solid tumours including breast [24], brain [25], prostate [26, 27], lung [28], colon [29], pancreas [30], liver [31], melanoma [32], skin, head, and neck [16]. The identification of CSCs in various malignancies has also revealed that CSCs are largely tissue specific and that a universal CSC marker is unlikely [17, 33, 34].

In addition to the abilities of self-renewal, differentiation, and regeneration, CSCs possess significant resistance to current treatment modalities such as chemotherapy and radiotherapy [35–44]. This characteristic of CSCs has significant treatment implications as current modalities allow resistant CSCs to reinitiate the tumour [45]. Current methods of reporting treatment success are based on measuring the overall reduction in the size and number of cells remaining after treatment [21, 46]. This does not take into account the proportion of cells within that population that are CSCs and therefore have the ability to orchestrate a relapse [21, 46]. The CSC theory demands a shift in measurement of treatment success [21, 46, 47]. Furthermore, it has become apparent that CSCs facilitate the metastatic characteristics of tumours [42, 48–54]. CSCs are capable of epithelial mesenchymal transition (EMT), which is a key step in wound healing and embryogenesis [33, 50, 55]. The genetic programs involved in EMT are activated in CSCs and involve breakdown of cell contacts and migration allowing the tumour cells to metastasize [33, 50]. The identification of this process and the biomarkers involved may serve as useful prognostic tools and therapeutic targets [50].

The isolation of CSCs via flow cytometry according to specific cell biomarkers is a widely used approach [56]. Dye exclusion assays have also been used to isolate side populations from tumour tissues. Finally, CSCs can be isolated via anchorage-independent culture assay. The identified cell must be able to “recapitulate the generation of a continuously growing tumor” [19]. As a result, the gold standard established for the qualification of this definition is a serial animal transplantation model [19]. Respect for the effect of the transplant and assay environments on the putative CSCs must be taken into consideration when assessing whether the cell meets the criteria of a CSC [19]. Studies have also been completed analysing the expression of “stemness” genes in suspected CSC populations using reverse transcriptase polymerase chain reaction (RT-PCR) techniques [19]. There is currently no ideal assay for the identification of CSCs however valuable insight and evidence of the role of CSCs in tumour initiation and progression are occurring.

This paper aims to provide a critical review of the putative HNSCC CSCs currently being investigated and provide evidence for potential novel markers.

## 3. CSC Biomarkers in HNSCC

### 3.1. CD44

CD44 is a large cell surface glycoprotein that is involved in cell adhesion and migration and is one of the most well-known markers for CSCs [13]. CD44 is thought to be involved in tumour progression and metastasis through its role as a regulator of growth, survival, differentiation, and migration [52, 57]. Through its interactions with hyaluronic acid (HA), chondroitin sulphate, and heparan sulphate, CD44 is able to bind growth factors and metalloproteinase MMP9, resulting in inhibition of apoptosis, collagen degradation, invasion, and neorovascularization [58–63]. CD44 has been identified as a biomarker in breast, CNS, colon, prostate, and pancreas tumours [16, 25, 26, 64–67]. Prince et al. used an immunodeficient mouse model to demonstrate that a subpopulation of cells derived from primary, unmanipulated HNSCC were able to meet two defining properties for a CSC: self-renewal and differentiation [16]. They were able to obtain new tumours from 5 × 10^3^ CD44^+^ cells derived from earlier passaged xenograft tumours, whereas 5 × 10^5^ CD44^−^ cells failed to form tumours. Despite demonstrating that the tumourigenic potential lay within the CD44^+^ cell population, the large number of cells needed to initiate tumours (5000) indicated that the CD44^+^ subpopulation was not a pure CSC population [16, 23]. Since then, several studies have demonstrated that CD44^+^ subpopulations, emanating from both primary tissues and cell lines, exhibit a higher potential for proliferation, differentiation, migration, invasion, tumour sphere formation, and resistance to chemotherapeutics [3, 5, 12, 16, 68–74]. It was also observed that CD44 isoforms v3, v6, and v10 were significantly associated with advanced primary tumour stage, metastasis, treatment failure, and reduced disease-free survival, indicating that CD44 is a useful marker for HNSCC progression and a possible target for therapy [75]. CD44 was used to assess the metastatic potential of HNSCC CSCs in both an in vitro and *in vivo* study showing no difference in metastatic ability in vitro but found CD44^high^ cells resulted in lung lesions, when injected in tails of NOD/SCID mice about 50% of the time compared to 0% for CD44^low^ cells [51]. Most recently, the frequency of CD44^+^ cells correlated with poor prognosis, more aggressive tumours, and higher rates of recurrence following radiotherapy [72]. These results further support the CSC theory and demonstrate that the CSC burden and not the overall burden is an important prognostic factor. The use of CD44 expression as a prognostic indicator has also been suggested, with several studies reporting a statistically significant association between CD44 expression and decreased 5-year survival [76, 77].

 Conversely, the use of CD44 as a marker has been questioned due to research suggesting that it is abundantly expressed in head and neck squamous cell carcinomas and that it is equally expressed in normal head and neck epithelium [50, 78]. A study conducted by Lim et al. (2011) also puts the use of CD44 as a CSC marker into question as they found that both CD44^+^ and CD44^−^ cells derived from squamospheres could regenerate these spheres from single cell suspensions [79].

### 3.2. ALDH

The aldehyde dehydrogenase family, of which ALDH1 is a member, is a family of cytosolic isoenzymes, which are highly expressed in many stem and progenitor cells [71, 80]. Their known functions include the conversion of retinol to retinoic acid in early stem cell differentiation and catalysing the oxidation of toxic intracellular aldehyde metabolites into carboxylic acid [71, 80]. As with CD44, the lead for investigating ALDH as a marker for CSCs in HNSCC followed identification in other solid malignancies such as breast, colon, liver, and lung tumours [81–84]. ALDH1^+^cells from head and neck squamous cell carcinoma cell lines and primary tissue samples have demonstrated spheroid formation, tumour formation, increased invasion capabilities, self-renewal abilities, and resistance to chemotherapeutics [42, 50, 71, 80, 85]. ALDH1 expression has a positive correlation to staging of HNSCC and a negative correlation to patient outcome [71]. As few as 500 ALDH1^+^ cells were able to generate tumours using an SCID mouse model, and ALDH1^+^ cells showed superior ability to form spheroid colonies, higher invasion capacity, and increased radiation survival [71, 80]. There is a significant overlap in the ALDH and CD44 populations with 50.6%–74.4% of ALDH1^+^ cells expressing CD44 and only 9.8%–23.6% of CD44 cells demonstrating high ALDH activity indicating that ALDH1 may be a more specific marker of CSCs in HNSCC [80]. Chen et al. further characterised the gene expression of ALDH1^+^cells and found the expression profile to be more similar to that of epithelial stem cells (ESCs) than ALDH1^−^ cells, detecting over expression of stemness-related genes and a reduction in cell-to-cell contact [50, 85]. As few as 20 ALDH1^+^/CD44^+^/CD24^−/low^ cells from breast cancer have been shown to initiate tumour growth; however such a small subpopulation capable of tumour growth is yet to be investigated in HNSCC [81]. Recent experiments have however found that low expression of ALDH is linked to poor prognosis rather than overexpression [86].

### 3.3. CD133

This pentaspan transmembrane glycoprotein has been identified as a putative CSC marker in brain, prostate, lung, skin, liver, and colorectal cancers [87]. A number of studies have suggested that CD133^+^ cells isolated from head and neck squamous cell carcinoma cell lines display increased clonogenicity, an EMT phenotype, tumour sphere formation, self-renewal, proliferation, multilinear differentiation, and tumorigenicity [69, 88–90]. Zhang et al. was able to isolate a CD133^+^ population of cells expressing higher levels of stemness genes, successful spheroid formation, heterogeneous tumour formation, and increased clonogenicity from OSCC cell lines (~1-2%) and also from human OSCC specimens (~1–3%) [88]. Recent studies have found a correlation between expression of CD133 and stage of carcinogenesis with stage III and IV tumours displaying higher levels than stages I and II. The role of CD133^+^ as a CSC marker in HNSCC still requires further investigation, and it is worth noting considerable discrepancy in CSC characteristics among other cancers in which CD133 is reported as a CSC marker with some studies showing similar tumour-initiating behaviour between CD133^+^ and CD133^−^ populations alike [91–94]. 

### 3.4. c-Met

c-Met, a tyrosine kinase receptor for hepatocyte growth factor (HGF), is associated with metastasis and tumour invasion, decreased survival, and was recently investigated as a marker for CSCs in HNSCC [54, 95, 96]. Sun and Wang found that 1000 c-Met^+^ cells demonstrated self-renewal and were able to generate heterogeneous tumours in 54.4% of cases and that 1000 c-Met^+^ cells were more tumourigenic than 1000 CD44^+^, which were only able to generate tumours in 33.3% of cases [54]. When both markers were used the success rate of the c-Met^+^/CD44^+^combination yielded tumours in 80% of cases injected with 1000 cells compared to the success rate of ALDH1^high^ cells, which yielded tumours in 66.6% of cases [54]. Unfortunately, not all HNSCC specimens were used in both transplantation phenotypes; however the specimens displayed an increase in tumour formation from 66.6% in the ALDH1^high^ to 75% in the c-Met^+^/CD44^+^ cells [54]. Further investigation with a greater number of samples and a comparison of c-Met^+^/CD44^+^ to c-Met^+^/CD44^+^/ALDH1^high^ tumourigenicity is yet to be completed.

### 3.5. Side Populations and Drug Efflux Transporters

Subpopulations of Hoechst 33342 dye-resistant cells termed “side population” (SP) cells have shown to express stem cell qualities when isolated from cancer samples [3]. It is thought that the dye is pumped out by the ATP-binding cassette (ABC) family of proteins including MDR1, MRP1, ABCB5, and ABCG2 [97]. SP cells from OSCC have shown to be more tumourigenic, chemoresistant and have demonstrated self-renewal *in vivo* [3]. Usually, Hoechst 33342 dye is effluxed by ABCG2 so it is considered to be a CSC marker in OSCC [3]. It has also been suggested that the presence of ABCG2 is strongly predictive of cancerous transformation of oral leukoplakia [98]. Similarly, high ABCB5 expression has shown to be associated with OSCC progression and recurrence making it a possible prognostic factor [99]. 

## 4. Stemness Markers

The use of CSC-isolating techniques such as sphere formation, side population, and flow cytometry has accommodated the profiling of these subpopulations for known stemness-related genes [3, 8, 42, 50, 85, 88]. These genes include transcription factors, that maintain pluripotency and self-renewal, genes related to EMT, quiescence, and components such as myofibroblasts that are engaged in maintaining the CSC stemness and niche.

It has been suggested that embryological stem cells have a core regulatory network involving three master regulators for self-renewal and maintenance of the undifferentiated state [100, 101]. These regulators are the POU domain transcription factor Oct-4 [102–104], the homeodomain transcription factor Nanog [105, 106], and the high mobility group protein Sox-2 [107]. Additionally, Oct-4 and Nanog have been suggested as two of the four major factors that allow reprogramming of adult cells into germ-line competent, induced pluripotent cells [108–110]. Oct-4 plays a critical role in the development and self-renewal of embryonic stem cells and has been linked to oncogenic processes [8, 111–113], Nanog maintains pluripotency of embryonic stem cells and functionally blocks differentiation [105, 114]. Chen et al. found that the epithelial stem cell gene Oct-4 was upregulated in ALDH1+ HNSCC cells and that Oct3/4, Sox2 and Nanog were all up regulated in a population of spheroid-derived cells from HNSCC [50, 85]. Similarly, Siu et al. demonstrated that Oct-3/4 along with TRA1-60, a tumour rejection antigen that is expressed on embryonic stem cells but vanishes upon differentiation, were detectable in the most invasive of six oral cancer cell lines suggesting that Oct-3/4 and TRA1-60 are markers indicating invasiveness [115]. Chiou et al. postulated that Oct-4 and Nanog play a role in tumour transformation, tumourgenicity, and metastasis finding an increased expression of these genes in a CSC-enriched subpopulation derived from sphere formation colonies from OSCC [8]. Furthermore, it was demonstrated that patients displaying a triple-positive expression of Oct-4, Nanog, and CD133 had the worst survival prognosis of all OSCC patients [8]. Additionally, it has been suggested that Oct-4 and Nanog overexpression is positively correlated with chemoresistance and stage while negatively correlated with differentiation status [8, 116]. This has led some to suggest that Oct-4 may be a useful prognostic indicator for hypopharyngeal squamous cell carcinoma [117]. 

SOX2 has shown to have increased expression (including copy number increase and amplification) in tumours of squamous lineage. It has been shown that SOX2 is amplified specifically in squamous cell carcinomas of the lung and esophagus but not in lung or esophageal adenocarcinomas [118]. This is thought to demonstrate SOX2 as a lineage-specific stem cell marker, and, in the context of its usefulness in identification of stem cells in HNSCC, this would be specifically related to the squamous lineage. Interestingly, however, a recent study found no change in expression of Oct-4, Nanog, or Sox2 between cell lines of varying degrees of disease from normal oral tissue to oral SCC [119].

EMT is an essential step in embryogenesis and wound healing allowing epithelial cells to breakdown cell-to-cell and cell-to-matrix connections facilitating the migration of these cells to other sites in the body and appears to be activated during epithelial cancers, facilitating migration and metastasis [49, 50, 55, 120, 121]. In order to metastasize, cancer cells undergo EMT and as such acquire the stem cell phenotype associated with this transformation [122]. The transcription factors Snail and Twist play key roles in the induction and coordination of EMT [123]. ALDH1+ HNSCC CSCs were found to have an increased expression of the Snail gene which correlated with metastasis, local recurrence, prognosis, proinflammatory mediators, and the aggressiveness of tumours [71, 85, 124–127]. Increased expression of Twist was found in CD44+ and ALDH+ HNSCC CSC-like cells and showed a loss of E-cadherin mediated cell-to-cell contacts, display of mesenchymal markers, and increased motility [70, 126]. The Wnt-signalling pathway is known to modulate neural crest development [128]. Abnormal Wnt signalling has already been demonstrated in HNSCC and may potentially induce Twist expression in carcinoma cells [126, 129, 130]. The EMT transcription factors ZEB1/2 have shown to be overexpressed in CSC-like cells in HNSCC and are linked to decreased survival rates, increased sphere formation, CD44+ cells, tumour growth, and metastasis [131]. 

These stem cell-related genes and the abnormal activation of the Wnt-signalling pathway in HNSCC provide valuable insight into the characteristics of CSCs and their role in metastasis, self-renewal, and maintaining pluripotency. Just how these genes relate to other CSC markers in head and neck cancers is still largely unknown; however these stemness genes may help to differentiate CSCs from normal tissue cells displaying similar markers.

### 4.1. Bmi-1

Bmi-1 is considered to be a stemness-related gene maintaining the self-renewal capacity of stem cells through regulating chromatin structure [7, 42, 85]. Although Bmi-1 has not been identified as a marker for HNSCC CSCs in its own right, its tumourigenic capacities and increased levels in ALDH1^+^ subpopulations have made it a target of examination and is considered a critical factor in predicting disease progression and clinical outcomes [42]. Bmi-1 is an essential constituent of the polycomb repressive complex 1, a key epigenetic regulator that regulates a number of biological processes, including X chromosome inactivation, carcinogenesis, and stem cell renewal [132, 133]. Through chromatin and histone modification Bmi-1 is believed to promote cellular proliferation [132, 133]. This is achieved by repressing the expression of the ink4a locus, which plays a pivotal role in the onset of cellular senescence. It also influences central tumour suppressors Rb and p53 [132, 133]. A moderate body of evidence has suggested that CSC-like subpopulations isolated from HNSCC cell lines and biopsies overexpress this protein [3, 16, 42, 85, 130, 134–136]. Insight into the role of Bmi-1 in such populations was provided by Chen et al. (2010), who used a lentiviral vector expressing sh-Bmi-1 to knock down Bmi-1 expression in an ALDH1^+^ HNSCC subpopulation [85]. Bmi-1 knock down resulted in significant suppression of self-renewal and radiochemoresistance both in vitro and *in vivo* [85]. Bmi-1 overexpression in a HNSCC-derived ALDH1^−^CD44^−^ subpopulation resulted in a restoration of stemness properties and self-renewal abilities and was sufficient for the promotion of CSC stemness [137]. Manufactured Bmi-1 overexpression in an ALDH1^+^-subpopulation of HNSCC resulted in increased soft agar colony formation, migration, invasion, and elevated expression of Snail, ALDH, and embryonic stem cell transcriptomes [42]. In addition, overexpression of Bmi-1 increases tumour formation, ionizing radiation resistance, local invasion, distant metastasis to the lungs, and tumour size *in vivo* [42, 138]. IHC analyses of HNSCC biopsies from 93 patients found that coexpression of Bmi-1, Snail and ALDH1 correlated with poor overall survival and was associated with high-grade, poorly differentiated HNSCC [42]. Bmi-1 may also play a role in the progression of potentially malignant lesions as it has been observed that Bmi-1 overexpression occurs in mild, moderate, and severe epithelial dysplasia [133]. It has also been suggested that the presence of Bmi-1 is strongly predictive of cancerous transformation of oral leukoplakic lesions [98]. 

Somewhat opposed to the above evidence, a study examining the correlation between Bmi-1 and prognosis of squamous cell carcinoma of the tongue has suggested that negative Bmi-1 immunoexpression, and not overexpression, was associated with a high risk of recurrence [132]. The divergence of tongue SCC from the above results could possibly relate to the varying pathophysiologies and aetiologies of HNSCC [42]. Some suggest that tongue cancer has a closer relationship with human papilloma virus as opposed to other aetiological agents such as tobacco and alcohol associated with other HNSCC [139, 140]. 

Despite the contradictions, it would appear that Bmi-1 plays a substantial role in the tumourigenesis of HNSCC. Future research is required to determine whether it is suitable as a CSC marker and the precise role it plays in the pathogenesis of HNSCC.

### 4.2. Lgr5

The Wnt/*β*-catenin-signalling pathway regulates proliferation and is central to embryonic development and organogenesis in several species. The main role of this pathway is to phosphorylate the *β*-catenin protein, leading to its protaesomal degradation. This is required as *β*-catenin interacts with DNA-binding protein transcription factor 4 leading to the activation of several genes [141, 142]. Abnormalities in this pathway, leading to cytoplasmic *β*-catenin accumulation, have been suggested to be associated with tumourigenesis in several tissues of epithelial origin including intestinal, colonic [142], esophageal [143], ovarian [144], and hepatic tissues [145]. Furthermore, it has been suggested that abnormal activation of this pathway is associated with head and neck carcinoma [130]. LGR5 (also known as GPR49) is a seven-transmembrane-domain receptor protein, and similar to EMT, is targeted by the Wnt-signalling pathway [146]. LGR5 marks rapidly dividing cells in hair follicles, colon, and the small intestine [146]. During embryogenesis, LGR5 has a broad and complex expression, and it has been suggested to be a marker for adult stem cells of the intestine, stomach, and hair follicle [147, 148]. LGR5 has been demonstrated as a marker for CSCs in colorectal cancer (CRC) and upregulated in oesophageal adenocarcinoma (EAC), basal cell carcinomas (BCCa) of the face, and cancers of the ovary and liver [143–146, 148–150]. Increased expression of LRG5 is correlated to poor survival rates in CRC and EAC and linked to increased proliferation and tumour formations in BCC [143, 146, 149]. A study by Morita et al. (2004) found LGR5 expression in the epithelium of the tongue and tissues of the mandible in wild-type mice and reported that knock down of these genes led to ankyloglossia, suggesting the importance of LGR5 in embryonic craniofacial development [151]. Interestingly, LGR5 has also been demonstrated as a marker for incisor stem cells in mice being found in the stellate reticulum compartment in the labial cervical loop [152]. Given that the Wnt/*β*-catenin-signalling pathway has been suggested to be abnormal in a side population of HNSCC cell lines and that LGR5 has been detected in the oral tissues of mice, further investigation into the effect of abnormal Wnt signalling in HNSCC on the expression of LGR5 and its candidature as a CSC marker in HNSCC may yield important insights into the behaviour of CSCs in HNSCC leading to superior prognostic and therapeutic strategies [129, 130].

### 4.3. Other Markers

Musashi-1 (Msi-1) is a translational regulator associated with both stem cell and tumour biology that has been recently correlated with OSCC and stage of carcinogenesis [153–155]. Cripto-1 is an extracellular, GPI-anchored signalling protein with important roles during embryonic development, stem cell function, and cancer progression [156–158]. Bone morphogenetic proteins (BMPs) play a diverse role in numerous biological processes [159, 160]. BMPs regulate proliferation, differentiation, and apoptosis during development and play roles in adult tissue maintenance, remodeling, and repair [161–164]. BMP-4 is thought to play roles in both cancer progression and inhibition in numerous human tumours [119, 165–182]. Chondroitin sulfate proteoglycan 4 (CSPG4) is a unique glycoprotein-proteoglycan complex that has been implicated in numerous aspects of melanoma cell biology and carcinoma (including HNSCC) with usefulness as a CSC in glioblastoma [183–189]. CXCR4 is a chemokine receptor that has been suggested to contribute to the metastasis of several cancers and has been found to be overexpressed in metastatic HNSCC [190–197]. CD166, present in all three embryological layers, is usually found in cells involved in growth and migration and has been implicated in cancers throughout the body indicating that research into its ability to identify CSCs at different stages of HNSCC may prove very useful [198]. SLC2A13 has been proposed as a putative marker for CSCs in OSCC after it was observed to be overexpressed in sphere formation derived from primary OSCC samples [199]. It has been suggested that podoplanin may be useful as a prognostic marker in the development and progression of head and neck cancer and as a biomarker for oral cancer risk in patients [165–176, 200–205]. A recent study of oral erythroplakia has suggested that podoplanin and ABCG2 expression may be beneficial for predicting progression to cancer as 90.9% of erythroplakic lesions expressing both markers became cancerous, while only 30% which expressed neither marker progressed [177]. 

## 5. Limitations and Implications

The limitations of current studies in this field are vast and for the most part generalised. This is due to the inherent nature of the research rather than research techniques. The main pitfall of the studies is their small sample size. This is due initially to the difficulty in obtaining primary tissue and further amplified by the even smaller population of CSCs that can be derived from these tissues. In addition to small sample size, most samples are drawn from varying sites of the head and neck region, TNM stage, gender, smoking status, alcohol use, age, and aetiology. Not only does the small sample size amplify these problems, but also it does not allow analysis of results to control for these variables. Although several researchers have attempted to use cell lines in order to bypass the problem of obtaining primary tumours, this does not solve the limitations mentioned and also introduces others. Studies using cell lines have only used a small number of different cell lines, which may not be representative of the total HNSCC population. Taking into account the heterogeneous nature of HNSCC and the potential evolution of the cells contained within these tumours, it seems that such samples are likely to be far from representative [69, 178]. It has been suggested that a tumour microenvironment or niche plays a large role in its behaviour including effects on growth stimulation, angiogenesis, and immunocompetence [14, 15, 179]. Cell lines effectively remove the tumour from this environment and establish an artificial one which has been constructed entirely to maintain the cell line, rather than to mimic the cell's original environment. The consequence of this confounder may be partially reduced by employing a human dermis-based organotypic culture model, such as the human oral mucosal equivalent (HOME) utilised by Dalley et al. [119, 180]. The problem with differing microenvironments also brings into question the current gold standard of CSC identification, mouse xenograft models [19]. Once again, the tumorigenic potential of a subset of HNSCC cells in a mouse microenvironment may not be representative of the natural niche of these cells. Currently, there is no single biomarker to define the CSC population accurately for HNSCC. Indeed, it seems a set of markers will be required to more narrowly define this population to achieve the best chance of developing targeted identification and treatment. The expression of the purported markers also needs further investigation over a longitudinal basis as expression of markers may evolve through interaction with the environment as the tumour progresses [181, 182]. Based on research to date, the number of cells needed to initiate tumours after sorting by CSC markers is much higher than in other solid tumours. Additionally, there are many markers that have been identified in other malignancies that have yet to be studied in HNSCC. 

The CSC hypothesis represents a fundamental shift in our understanding of the nature and pathogenesis of cancer. This insight requires reevaluation of how we diagnose develop prognoses and effective therapies and how the effectiveness of those therapies are assessed. According to the now widely supported CSC theory, just one CSC may be capable of initiating and progressing the full heterogeneity of a tumour, whereas many non-CSCs cannot. The need for more specific therapeutic targets has emerged, redirecting efforts from eliminating the bulk of differentiated cells from a tumour to the smaller minority of CSCs. Therapies targeting CSCs must overcome the chemoresistance, radioresistance, and immune evasion mechanisms of CSCs [33]. Biomarkers for HNSCC CSCs can hopefully play an integral role in not only understanding their role in the “stemness” of CSCs, but also as possible targets for therapy themselves. Targeted therapy is likely to have a higher therapeutic index and therefore less toxicity than current, less specific treatments [43]. As well as the biomarkers themselves, their signalling pathways and products may serve as targets for their elimination [17]. Another possibility is using nanotechnologies that are able to bypass the efflux mechanisms of these proteins [17, 206]. The Bmi-1 gene has shown to play an important role in chemo- and radioresistance in HNSCC CSCs, with knock down of the gene in HNSCC ALDH1^+^ cells inhibiting tumourigenicity and enhancing chemo- and radiosensitivity [42, 85]. The inhibition of binding of CD44 with HA interferes with important steps in tumour development such as inhibition of apoptosis, invasion, and angiogenesis; however the similar prevalence of CD44 in normal and malignant tissues alike highlights the need for ideal therapies to target CSCs while sparing normal stem cells [52]. As with normal stem cells, CSCs may be quiescent until stimulated by the microenvironment; elucidation of this interaction may also provide more specific targets for therapy. HNSCC therapy may also be complicated by the fact that a subpopulation of cells driving the tumour at one point in time may not be the same group at another point in time, indicating a need for longitudinal characterisation of CSCs to further refine treatments [19]. Further understanding of the similarities and differences in biology and resistance mechanisms of normal stem cells and cancer stem cells is needed to develop efficient and effective novel HNSCC therapies. Indeed survival studies need to take into account defined patient subpopulations when reporting correlation of biomarkers with patient survival. A more defined analysis of CSC markers in HNSCC subpopulations undergoing adjuvant therapy should be undertaken to more clearly outline the clinical relationship and usefulness of CSC markers and response to HNSCC therapy.

## 6. Summary

The discovery of CSCs has heralded an exciting era in our understanding of HNSCC with significant implications for diagnosis, prognosis, treatment, and ultimately patient outcomes. Currently, there is no single biomarker to define the CSC population accurately for HNSCC. Indeed, it seems a set of markers will be required to more narrowly define this population to achieve the best chance of developing targeted identification and treatment. The expression of purported markers needs further investigation over a longitudinal basis as expression of markers may evolve through interaction with the environment as the tumour progresses. Based on research to date, the number of cells needed to initiate tumours after sorting by CSC markers is much higher than in other solid tumours. Additionally, there are many markers that have been identified in other malignancies that have yet to be studied in HNSCC. Identification of biomarkers for HNSCC CSCs is only the first step in uncovering the nature of the disease as further investigation into the roles and mechanisms of these markers is ultimately needed to provide understanding of the pathogenesis and direction for future treatment. It is clear that further research to define the set of biomarkers for this population of cells is required to ultimately achieve superior detection, diagnosis, prognosis, and treatment outcomes in HNSCC patients.
